# Supplementary material for: Extraembryonic gut endoderm cells undergo programmed cell death during development
Source: Nat Cell Biol. 2024 Jun 7;26(6):868–77. doi: 10.1038/s41556-024-01431-w (PMC11178501; doi:10.1038/s41556-024-01431-w)
Supplement: Supplementary file 1 — Supplementary Figs. 1–3. [file 41556_2024_1431_MOESM1_ESM.pdf]

nature cell biology

Supplementary information

https://doi.org/10.1038/s41556-024-01431-w

Extraembryonic gut endoderm cells undergo programmed cell death during development

In the format provided by the authors and unedited

Representative flow cytometry gating strategies for E9.5 embryos and the posterior halves

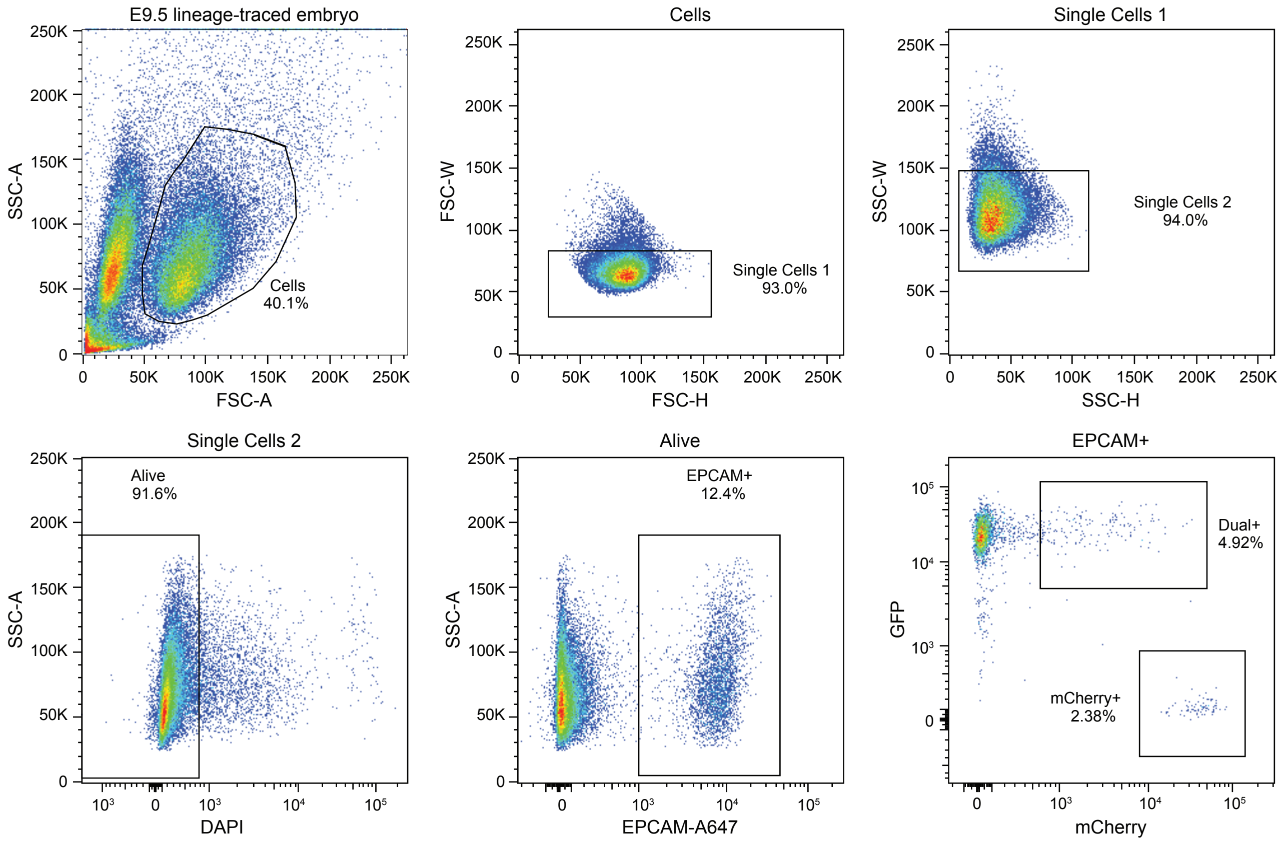

Representative flow cytometry gating strategies for E13.5 organs

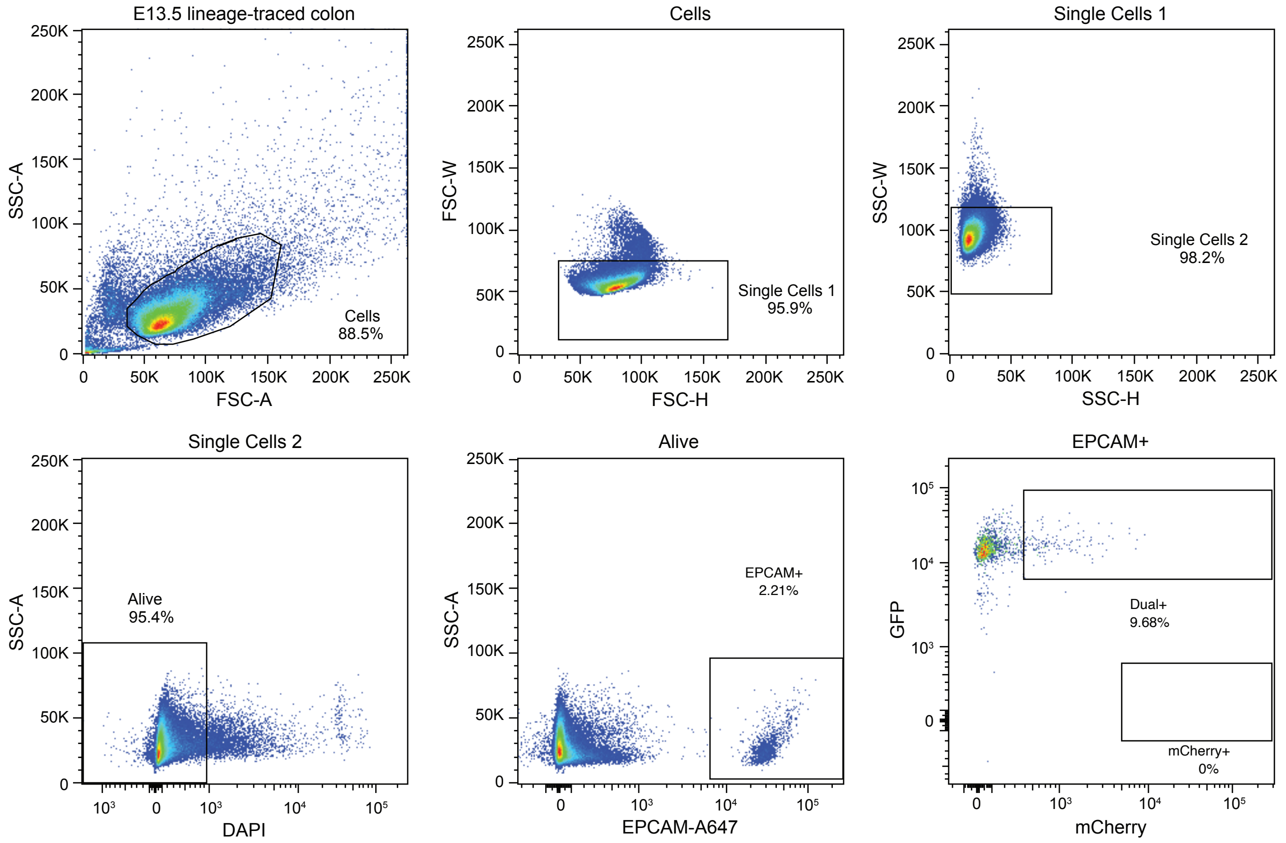

Supplementary Figure 1

Representative flow cytometry gating strategies for E9.5 midgut and hindgut

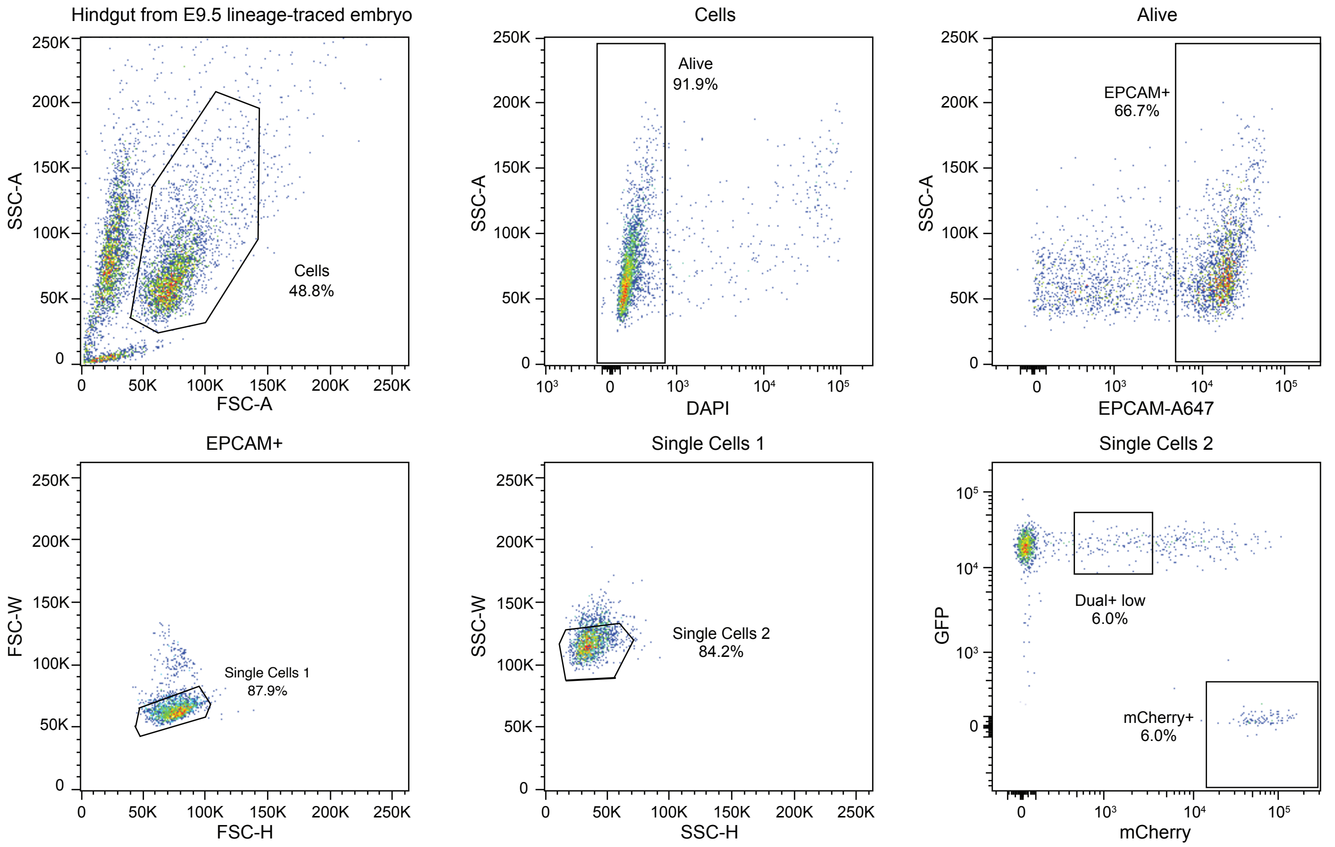

Representative flow cytometry gating strategies for E9.5 yolk sac endoderm

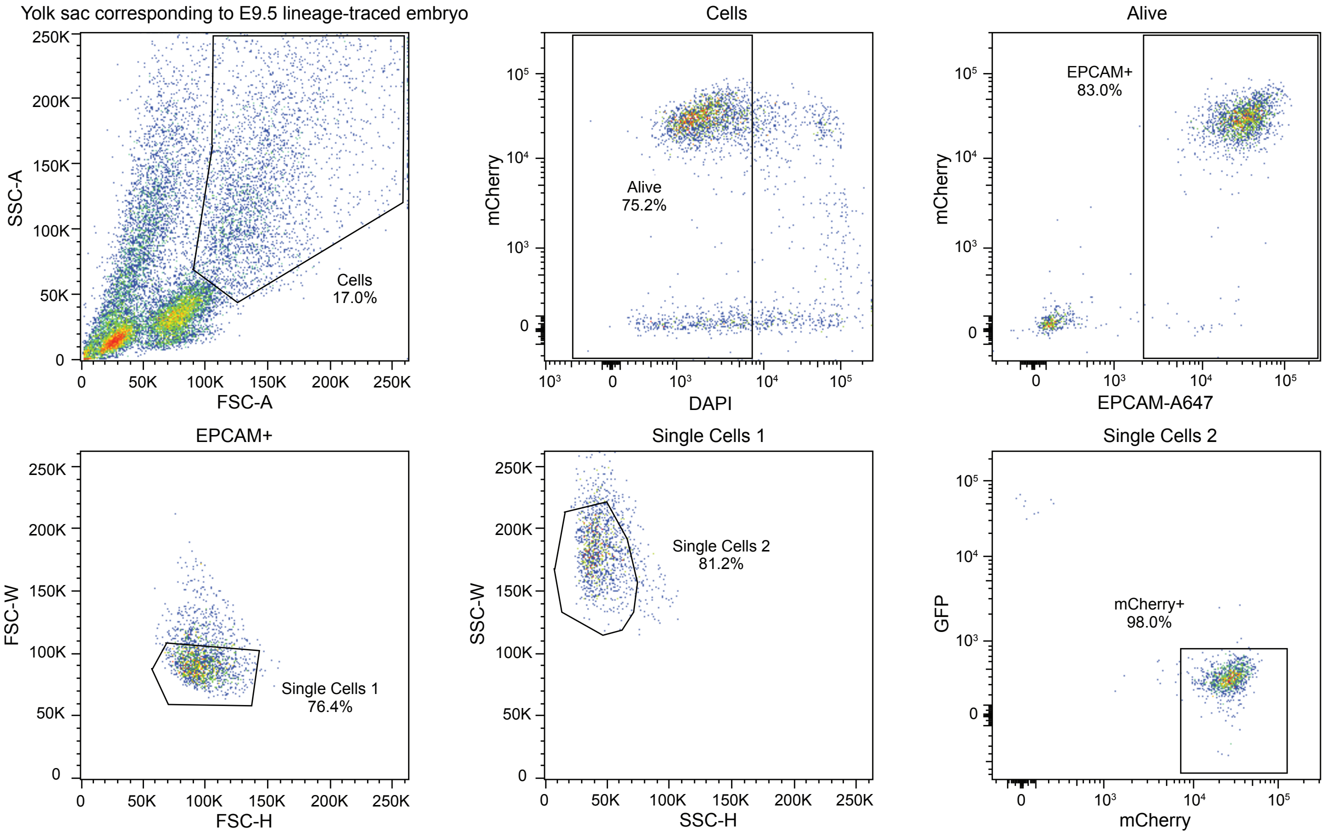

Supplementary Figure 2

Representative flow cytometry gating strategies for E13.5 gastrointestinal tract

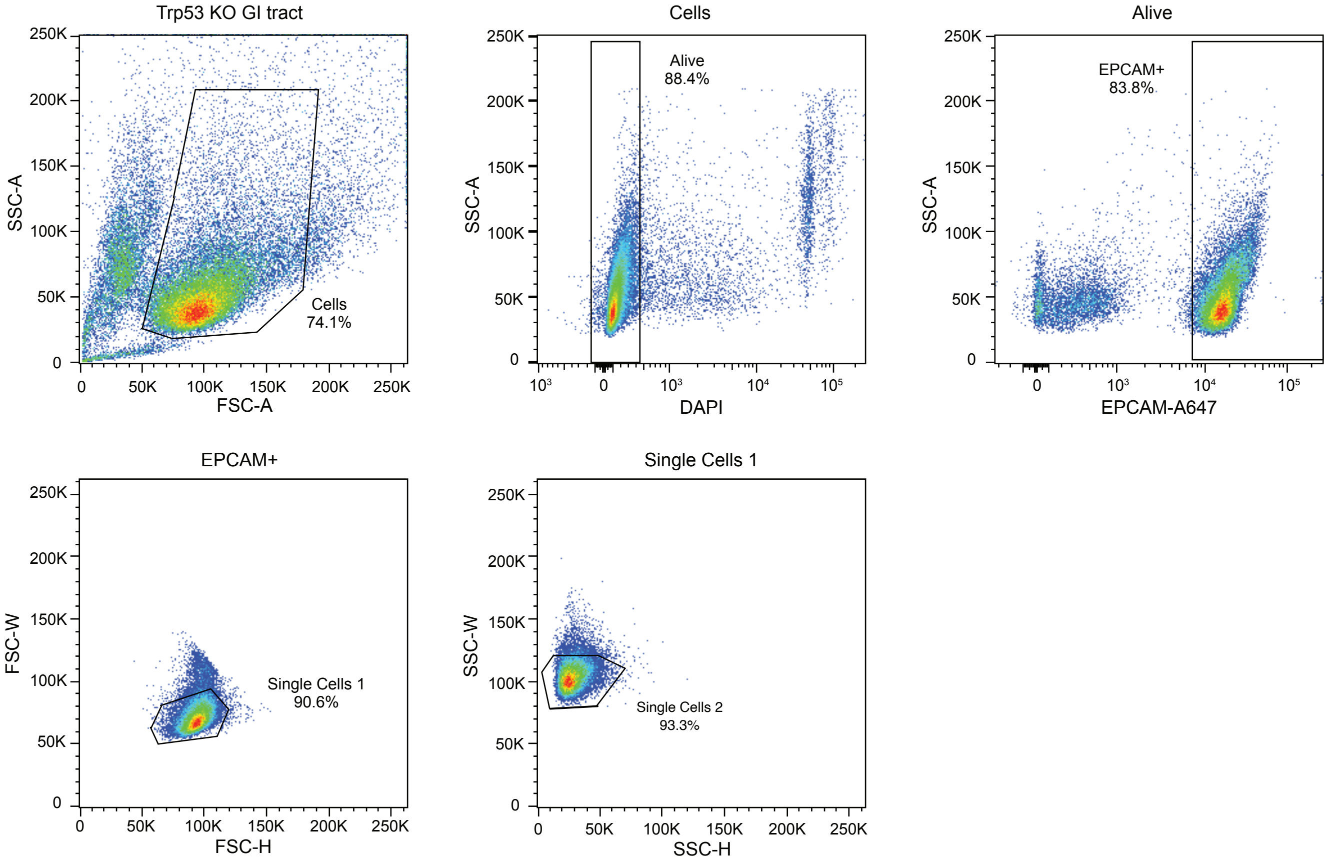

Supplementary Figure 3
