# Supplementary material for: Extraembryonic gut endoderm cells undergo programmed cell death during development
Source: Nat Cell Biol. 2024 Jun 7;26(6):868–77. doi: 10.1038/s41556-024-01431-w (PMC11178501; doi:10.1038/s41556-024-01431-w)
Supplement: Supplementary file 2 — Reporting Summary [file 41556_2024_1431_MOESM2_ESM.pdf]

nature portfolio

Corresponding author(s): Alexander Meissner

Last updated by author(s): Apr 2, 2024



# Reporting Summary

Nature Portfolio wishes to improve the reproducibility of the work that we publish. This form provides structure for consistency and transparency in reporting. For further information on Nature Portfolio policies, see our Editorial Policies and the Editorial Policy Checklist.

## Statistics

For all statistical analyses, confirm that the following items are present in the figure legend, table legend, main text, or Methods section.

| n/a | Confirmed | |
|---|---|---|
| ☐ | ☒ | The exact sample size (*n*) for each experimental group/condition, given as a discrete number and unit of measurement |
| ☐ | ☒ | A statement on whether measurements were taken from distinct samples or whether the same sample was measured repeatedly |
| ☐ | ☒ | The statistical test(s) used AND whether they are one- or two-sided<br>*Only common tests should be described solely by name; describe more complex techniques in the Methods section.* |
| ☐ | ☒ | A description of all covariates tested |
| ☐ | ☒ | A description of any assumptions or corrections, such as tests of normality and adjustment for multiple comparisons |
| ☒ | ☐ | A full description of the statistical parameters including central tendency (e.g. means) or other basic estimates (e.g. regression coefficient) AND variation (e.g. standard deviation) or associated estimates of uncertainty (e.g. confidence intervals) |
| ☐ | ☒ | For null hypothesis testing, the test statistic (e.g. *F*, *t*, *r*) with confidence intervals, effect sizes, degrees of freedom and *P* value noted<br>*Give P values as exact values whenever suitable.* |
| ☒ | ☐ | For Bayesian analysis, information on the choice of priors and Markov chain Monte Carlo settings |
| ☒ | ☐ | For hierarchical and complex designs, identification of the appropriate level for tests and full reporting of outcomes |
| ☒ | ☐ | Estimates of effect sizes (e.g. Cohen's *d*, Pearson's *r*), indicating how they were calculated |

*Our web collection on statistics for biologists contains articles on many of the points above.*

## Software and code

Policy information about availability of computer code

| | |
|---|---|
| Data collection | 10x Genomics Cell Ranger (version 6.0.1), deMULTIplex (version 1.0.2), cutadapt (version 4.1), STAR (version 2.7.9a), stringtie (version 2.0.6), BSMAP (version 2.90), MOABS (version 1.3.2), GATK (version 4.3.0.0), BD FACS Diva (version 8.0.1), Zeiss ZEN Blue (version 3.5), ZEN 2014 |
| Data analysis | R (version 4.1.0), Seurat (version 4.1.0), DESeq2 (version 1.32.0), WebGestaltR (version 0.4.4), ComplexHeatmap (version 2.7.11), bedtools (version 2.30.0), metilene (version 0.2-8), IGV (version 2.15.2), Zeiss ZEN Black (version 2.3 SP1 FP3), ImageJ2 (version 2.3.0/1.53q), FlowJo (version 10.8.1), samtools (version 1.18), deeptools (version 3.5.2)<br><br>Custom code is available at https://doi.org/10.5281/zenodo.10926934. |

For manuscripts utilizing custom algorithms or software that are central to the research but not yet described in published literature, software must be made available to editors and reviewers. We strongly encourage code deposition in a community repository (e.g. GitHub). See the Nature Portfolio guidelines for submitting code & software for further information.

## Data

Policy information about availability of data

All manuscripts must include a data availability statement. This statement should provide the following information, where applicable:

- Accession codes, unique identifiers, or web links for publicly available datasets
- A description of any restrictions on data availability
- For clinical datasets or third party data, please ensure that the statement adheres to our policy

Sequencing data sets generated within the scope of this study have been deposited in the Gene Expression Omnibus under accession no. GSE250084. scRNA-seq data sets of E8.75 gut endoderm and E9.5-E15.5 gastrointestinal tract were obtained from GSE123046 and GSE186525, respectively. WGBS data sets of wild type E6.5 epiblast were obtained from GSE137337. The mouse reference genome mm10 was obtained from UCSC (https://hgdownload.soe.ucsc.edu/goldenPath/mm10/bigZips/). Annotations of CpG islands for mm10 were downloaded from UCSC (https://genome.ucsc.edu/cgi-bin/hgTables). The mm10 gene annotation was downloaded from GENCODE (VM23, https://www.gencodegenes.org/mouse/release_M23.html). Source data are provided at https://doi.org/10.5281/zenodo.10926934. All other data supporting the findings of this study are available from the corresponding author on reasonable request

## Human research participants

Policy information about studies involving human research participants and Sex and Gender in Research.

| | |
|---|---|
| Reporting on sex and gender | N/A |
| Population characteristics | N/A |
| Recruitment | N/A |
| Ethics oversight | N/A |

Note that full information on the approval of the study protocol must also be provided in the manuscript.

# Field-specific reporting

Please select the one below that is the best fit for your research. If you are not sure, read the appropriate sections before making your selection.

☒ Life sciences ☐ Behavioural & social sciences ☐ Ecological, evolutionary & environmental sciences

For a reference copy of the document with all sections, see nature.com/documents/nr-reporting-summary-flat.pdf

# Life sciences study design

All studies must disclose on these points even when the disclosure is negative.

| | |
|---|---|
| Sample size | No statistical methods were used to predetermine sample sizes but our sample sizes are similar to those reported in previous publications (Auclair et al. Genome Biology 2014, Nowotschin et al. Nature 2019, Grosswendt et al. Nature 2020, Scheibner et al. Nature Cell Biology 2021, Rothová et al. Nature Cell Biology 2022). Sample sizes are indicated in the figure panels or legends. |
| Data exclusions | Prior to downstream analysis and experiments, resorping embryos were excluded. For the downstream experiments with the two-color lineage-tracing, only embryos with gut-specific mCherry+ signal were used, mCherry+ only embryos were excluded. No other data was excluded. |
| Replication | For RNAseq and RRBS experiments three to four replicates were generated. For E9.5, embryos were pooled while for E6.5 and E13.5, single embryo replicates were generated. For WGBS experiments, two replicates were generated for each E6.5 tissue (exEndo 1 and 2) and one replicate was generated for each E9.5 tissue. For the E9.5 scRNA-seq analysis, one experiment was performed, which contained cells of different sort groups (dual+ low, intermediate and high populations, mCherry+ population) from 15 pooled embryos. For the E13.5 scRNA-seq analysis, four WT embryos and four Trp53 knockout embryos were included in the experimental set-up labeled by MULTI-seq barcodes, which allowed comparison of cell state distributions across single embryo replicates. For imaging experiments and FACS analysis, 3-10 embryos were analyzed, the exact number is indicated in the respective figure or legend. All attempts at replication were successful. |
| Randomization | For assessing the outcome of the complementation assays, embryos were collected without a preconceived selection strategy or prioritization by morphology. Our genomic analyses are independent of human intervention and analyze each sample equally and in an unbiased fashion. |
| Blinding | Data collection and analysis were not performed blind to the conditions of the experiments. Blinding was not relevant for this study since this is not an intervention study. However, our analytical pipeline followed uniform criteria applied to all samples, allowing us to analyse our data in an unbiased manner. |

# Reporting for specific materials, systems and methods

We require information from authors about some types of materials, experimental systems and methods used in many studies. Here, indicate whether each material, system or method listed is relevant to your study. If you are not sure if a list item applies to your research, read the appropriate section before selecting a response.

## Materials & experimental systems

| n/a | Involved in the study | |
|---|---|---|
| ☐ | ☒ | Antibodies |
| ☐ | ☒ | Eukaryotic cell lines |
| ☒ | ☐ | Palaeontology and archaeology |
| ☐ | ☒ | Animals and other organisms |
| ☒ | ☐ | Clinical data |
| ☒ | ☐ | Dual use research of concern |

## Methods

| n/a | Involved in the study | |
|---|---|---|
| ☒ | ☐ | ChIP-seq |
| ☐ | ☒ | Flow cytometry |
| ☒ | ☐ | MRI-based neuroimaging |

## Antibodies

Antibodies used

Primary antibodies: Foxa2 (HNF-3β) antibody (Santa Cruz, sc-6554, 1:250 dilution), E-cadherin antibody (Cell Signaling Technology, 3195, 1:250 dilution), cleaved-Caspase3 antibody (Cell Signaling Technology, 9661, 1:250 dilution), mCherry antibody (abcam, ab167453, 1:200 dilution), mCherry antibody (antibodies-online, ABIN1440058, 1:500 dilution), Epcam (CD326) antibody Alexa Fluor 647 (BioLegend, 118211, 1:400 dilution)
Secondary antibodies: Alexa Fluor 647 Donkey anti-Goat (Invitrogen, A21447, 1:250 dilution), Alexa Fluor 647 Donkey anti-Rabbit (Invitrogen, A31573, 1:250 dilution), Alexa Fluor 546 Donkey anti-Goat (Invitrogen, A11056, 1:250 dilution), Alexa Fluor 568 Donkey anti-Rabbit (Invitrogen, A10042, 1:250 dilution)

Validation

All antibodies were validated by their manufacturers:
Foxa2 antibody: https://www.scbt.com/p/hnf-3beta-antibody-m-20?productCanUrl=hnf-3beta-antibody-m-20&_requestid=2453355
E-cadherin antibody: https://www.cellsignal.de/products/primary-antibodies/e-cadherin-24e10-rabbit-mab/3195
cleaved-Caspase3 antibody: https://www.cellsignal.de/products/primary-antibodies/cleaved-caspase-3-asp175-antibody/9661
mCherry antibody: https://www.abcam.com/mcherry-antibody-ab167453.html
mCherry antibody: https://www.antibodies-online.com/antibody/1440058/anti-mCherry+Fluorescent+Protein+antibody/
Epcam (CD326) antibody: https://www.biolegend.com/en-us/search-results/alexa-fluor-647-anti-mouse-cd326-ep-cam-antibody-4973?GroupID=BLG5748
lexa Fluor 647 Donkey anti-Goat: https://www.thermofisher.com/antibody/product/Donkey-anti-Goat-IgG-H-L-Cross-Adsorbed-Secondary-Antibody-Polyclonal/A-21447
Alexa Fluor 647 Donkey anti-Rabbit: https://www.thermofisher.com/antibody/product/Donkey-anti-Rabbit-IgG-H-L-Highly-Cross-Adsorbed-Secondary-Antibody-Polyclonal/A-31573
Alexa Fluor 546 Donkey anti-Goat: https://www.thermofisher.com/antibody/product/Donkey-anti-Goat-IgG-H-L-Cross-Adsorbed-Secondary-Antibody-Polyclonal/A-11056
Alexa Fluor 568 Donkey anti-Rabbit: https://www.thermofisher.com/antibody/product/Donkey-anti-Rabbit-IgG-H-L-Highly-Cross-Adsorbed-Secondary-Antibody-Polyclonal/A10042

## Eukaryotic cell lines

Policy information about cell lines and Sex and Gender in Research

Cell line source(s)

The cell lines used in this study (mCherry+ mESCs and GFP+ mESCs) were generated in house, are male and derived from an F1G4 genetic background (George et. al., 2007), which was obtained from the laboratory of A. Nagy.

Authentication

None of the transgenic cell lines generated in this study have been authenticated.

Mycoplasma contamination

All cell lines used in this study tested negative for mycoplasma contamination.

Commonly misidentified lines
(See ICLAC register)

No commonly misidentified cell lines were used in this study.

## Animals and other research organisms

Policy information about studies involving animals; ARRIVE guidelines recommended for reporting animal research, and Sex and Gender in Research

Laboratory animals

All animal work performed in this study was approved by the local authorities (LAGeSo Berlin, license number G0243/18, G0098/23). Mice were kept in individually ventilated cages (IVC) under specified pathogen free (SPF) conditions in animal rooms with a light cycle of 12h/12h, a temperature of 20-24°C and a humidity of 45-65%. The mice received autoclaved water and a standard rodent diet ad libidum. For embryo generation, Hsd:ICR (CD-1) females (age 6-20 weeks) were mated with the indicated males (CD-1 or mCherry+) (age ≥8weeks). Blastocysts resulting from complementation assays were transfered into CD-1 foster females (age 6-20 weeks) that

| | |
|---|---|
| | were previously mated to vasectomized CD-1 males (age ≥12 weeks) to induce pseudopregnancy. Hsd:ICR (CD-1) and C57BL/6J animals were obtained from Envigo/Inotiv. |
| Wild animals | The study did not involve wild animals. |
| Reporting on sex | GFP+ embryos originating from the lineage tracing assay are male because the mESC line used in the complementation assay is male. mCherry+ pre-implantation embryos were generated via natural mating, resulting in both male and female cells. |
| Field-collected samples | No field-collected samples were used in this study. |
| Ethics oversight | All research described here complies with the relevant ethical regulations and was approved by the LAGeSo Berlin, license number G0243/18 and G0098/23. |

Note that full information on the approval of the study protocol must also be provided in the manuscript.

# Flow Cytometry

## Plots

Confirm that:

☒ The axis labels state the marker and fluorochrome used (e.g. CD4-FITC).

☒ The axis scales are clearly visible. Include numbers along axes only for bottom left plot of group (a 'group' is an analysis of identical markers).

☒ All plots are contour plots with outliers or pseudocolor plots.

☒ A numerical value for number of cells or percentage (with statistics) is provided.

## Methodology

| | |
|---|---|
| Sample preparation | Deciduae were collected into ice-cold HBSS (Gibco #14175095), E9.5 embryos (somite number 18-28) were dissected in ice-cold M2 medium (Merck #MR-015-D), the extraembryonic tissues were completely removed, and the yolk sac was kept. For the single-cell RNA-seq analysis to determine the cell type identities of mCherry+ and dual+ cells, whole lineage-traced embryos were used. For assessing extraembryonic cell content in lineage-traced embryos (comparing wild type and p53 extraembryonic-specific knockout), the embryos were cut into two halves with a micro knife along the anterior-posterior axis, and the posterior half was used further. For RNA-seq, RRBS, and WGBS experiments, wild type lineage-traced E9.5 embryos were cut into two halves with a micro knife along the anterior-posterior axis. From the posterior half, the midgut was manually isolated using tungsten needles (Fine Science Tools #10130-10), and the most posterior part was also kept containing the hindgut. For each midgut and hindgut replicate, corresponding tissues from four embryos were pooled.<br>The embryos, the isolated tissues, and the yolk sac were washed in ice-cold HBSS, dissociated with 0,25 % Trypsin-EDTA (Gibco #25200056) for 10 minutes at 37°C to obtain single cells. This was quenched with KnockOut DMEM (Thermo Fisher Scientific #10829018) with 10% FBS (PAN-Biotech #P30-2602) and 0,05 mg/ml DNase I (Merck #11284932001) to dissociate the cells via pipetting, and the cells were also washed once with this buffer. After blocking with Normal Mouse Serum (Invitrogen #31881) for 5 minutes on ice, cells were stained for EPCAM (Alexa Fluor® 647 anti-EPCAM, BioLegend #118212) in FACS buffer (HBSS with 2% FBS and 0,5 mM EDTA (Thermo Fischer Scientific #15575020)) for 10 minutes on ice. Specifically for the pooled midgut and hindgut samples, enrichment of EPCAM+ cells was performed by magnetic separation (MACS) using Anti-Cy5/Anti-Alexa Fluor 647 MicroBeads (Miltenyi Biotec #130-091-395), following the manufacturer's instructions with the MS columns (Miltenyi Biotec #130-042-201) and the OctoMACS™ Separator (Miltenyi Biotec #130-042-109). Last, cells were stained with DAPI (0.02 %, Roche Diagnostics #102362760019) in FACS buffer for 8 minutes on ice, then were washed once and resuspended in FACS buffer, and kept on ice until flow cytometry analyses or sorting.<br>E13.5 embryos were dissected in DMEM/F-12 (Thermo Fischer Scientific #21041025) with 10 % FBS (PAN-Biotech #P30-2602). For scRNA-seq analysis of the wild type and p53 knockout embryos (see below), the gastrointestinal tract was isolated, then dissociation, staining for EPCAM, enrichment by MACS, and preparation for FACS was performed as described above for E9.5 midgut and hindgut samples. For RNA-seq and RRBS, E13.5 lineage-traced embryos were collected, and the intestine was isolated, and split into the small intestine and colon parts with a micro knife. Then, dissociation, staining for EPCAM, and sample preparation for FACS were performed as described above for E9.5 embryos. |
| Instrument | BD FACS ARIA FUSION (Becton Dickinson) |
| Software | DIVA, FlowJo (v10.8.1). |
| Cell population abundance | Given the low input for our sorting experiments, sort check was not performed on the sorted material, instead separate samples of the same type were used to sort the desired populations and perform post-sort checks which confirmed the purity of the sort-test sample. |
| Gating strategy | For analysis of extraembryonic gut cell content in embryos and organs, the following gating strategy was set up. First, an FSC-A vs SSC-A gating was used to identify the cell population. Next, two doublet removal steps were performed (FSC-W vs FSC-H and SSC-W vs SSC-H). Alive cells were gated based on DAPI, then epithelial/endoderm cells were gated based on EPCAM. For sorting gut endoderm and yolk sac endoderm cells, the following strategy was used. First, an FSC-A vs SSC-A gating was used to identify the cell population. Alive cells were gated based on DAPI, then epithelial/endoderm cells were gated based on EPCAM. Next, two doublet removal steps were performed (FSC-W vs FSC-H and SSC-W vs SSC-H). Finally, cells were sorted based on GFP and mCherry intensities. For sorting endoderm cells from the gastrointestinal tracts, the following strategy was used. First, an FSC-A vs SSC-A gating was used to identify the cell population. Alive cells were gated based on DAPI, then epithelial/endoderm cells were gated based on EPCAM. Next, two doublet removal steps were performed (FSC-W vs FSC-H |

and SSC-W vs SSC-H) and single cells were sorted. Representative gating strategies are provided in Supplementary Information 1, and example plots are also provided in the Figures and Extended Data Figures.

☒ Tick this box to confirm that a figure exemplifying the gating strategy is provided in the Supplementary Information.
